# The Significance of Exosomal RNAs in the Development, Diagnosis, and Treatment of Gastric Cancer

**DOI:** 10.3390/genes12010073

**Published:** 2021-01-08

**Authors:** Guiping Zhao, Anni Zhou, Xiao Li, Shengtao Zhu, Yongjun Wang, Shutian Zhang, Peng Li

**Affiliations:** Department of Gastroenterology, Beijing Friendship Hospital, Capital Medical University, Beijing 100050, China; zhaoguiping@ccmu.edu.cn (G.Z.); pineappleanni@163.com (A.Z.); lxaigni@163.com (X.L.); zhushengtao@ccmu.edu.cn (S.Z.); wyj_30302@163.com (Y.W.); zhangshutian@ccmu.edu.cn (S.Z.)

**Keywords:** exosome, exosomal RNAs, gastric cancer, biomarker

## Abstract

Gastric cancer (GC) is one of the most common malignancies in the world. Exosomes, a subset of extracellular vesicles with an average diameter of 100 nm, contain and transfer a variety of functional macromolecules such as proteins, lipids, and nucleic acids. A large number of studies indicated that exosomes can play a significant role in the initiation and development of GC via facilitating intercellular communication between gastric cancer cells and microenvironment. Exosomal RNAs, one of the key functional cargos, are involved in the pathogenesis, development, and metastasis of GC. In addition, recent studies elucidated that exosomal RNAs may serve as diagnostic and prognostic biomarkers or therapeutic targets for GC. In this review, we summarized the function of exosomal RNA in the tumorigenesis, progression, diagnosis, and treatment of GC, which may further unveil the functions of exosome and promote the potentially diagnostic and therapeutic application of exosomes in GC.

## 1. Introduction 

Gastric cancer (GC) is the fourth most common malignance and the third leading cause of cancer death among males worldwide [1]. The estimated new cases and deaths of GC every year is about 951,600 and 723,100 [2]. The incidence rate of GC in males is about twice than in females and varies wildly among countries, and more than half of those cases occur in Eastern Asia. *Helicobacter pylori* (*Hp*) infection is considered the most important risk factor for GC and over 90% of the non-cardia gastric cancers are related to *Hp* infection [3]. Thanks to the advances in the early diagnosis and treatment of GC, a steady decrease of GC cases has been observed in most developed countries, but the situation in many developing countries is still far from satisfactory. Due to the shortage of regular screening, improved sanitation, and effective antibiotics, the early diagnosis rate and prognosis of patients in developing countries remains poor. To date, the overall five-year survival rate of advanced GC after surgery is less than 30% and the five-year survival rate of early GC after Endoscopic submucosal dissection (ESD) is over 90% [4,5,6,7], thus revealing the specific formation mechanisms and improving the early diagnostic rate of GC is of vital importance.

Exosomes, initially introduced in 1980s by Trams, are a subset of extracellular vesicles (EVs) with an average diameter of 100 nm [8]. Further studies found that exosomes contain a variety of bioactive molecules including proteins, lipids, mRNAs, and noncoding RNAs (ncRNAs) and play a significant role in intercellular crosstalk [9]. Exosomal contents can be internalized by the recipient cell and mediate the activity of recipient cells, thereby functioning as a vehicle of cell-cell communication and are involved in many physiological and pathological activities including mammalian reproduction and development, immune responses and infection, cardiovascular diseases and cancers [10,11,12,13,14]. In the course of tumor development, exosomes not only facilitate the formation of a tumor microenvironment (TME) but also play a role in the occurrence, proliferation, metastasis, angiogenesis, and drug resistance. Additionally, it has been identified that the amounts of secreted exosomes and the exosomal compositions in cancer patients was different compared with healthy donors and the contents and quantity of exosomes secreted by the same cell can also be sharply different when treated with different conditions [15,16,17]. Theoretically, the contents of tumor cell-derived exosomes (TDEs) may include tumor-related biomarkers, such as micro RNAs (miRNAs), long non-coding RNAs (lncRNAs), and circular RNAs (circRNAs) or proteins, which may be detected in the early stage of GC and serve as noninvasive biomarkers for early detection and diagnosis of GC. Moreover, recent studies consider exosomes as ideal therapeutic targets and exosome-based therapies are emerging as a promising strategy for GC treatment [18,19]. In this review, we summarize the functions of exosomal RNA in the tumorigenesis, progression, diagnosis, and treatment of GC, which may further unveil the secrets of exosome and promote the potentially diagnostic and therapeutic application of exosome in GC.

## 2. Exosome Formation, RNAs Packaging and Uptake

**Figure 1:**
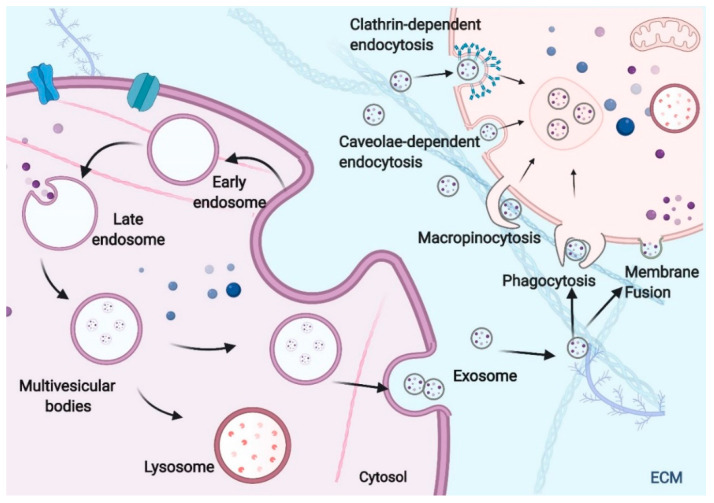
The biogenesis and uptake of exosomes. The invagination of the cell plasma membrane forms the early endosome, which embodies cell surface proteins and soluble proteins in extracellular space. Then, the early endosomes can mature into late endosomes, and invagination of the endosomal membrane results in the formation of multivesicular bodies (MVBs). This process allows the cytoplasmic constituents to enter the intraluminal vesicles (ILVs). The MVB can either be degraded by lysosomes or fuse with the cell membrane to secrete ILVs as exosomes. Exosomes from the parent cell can dock at and enter the recipient cell via several mechanisms, including fusion with the plasma membrane, clathrin-dependent endocytosis, caveolae-dependent endocytosis, macropinocytosis, and phagocytosis.

Exosomes are small, membrane-encapsulated vesicles 30–200 nm in diameter that are enriched in selected proteins, lipids, mRNAs, and miRNAs [20]. Generally, exosomes are formed by the invagination of late endosomal membranes and sequential engulfment of molecular materials in cytoplasm to form multivesicular bodies (MVBs), followed by fusing MVBs with the cell membrane to release intraluminal vesicles (ILVs) [21]. The inward invagination of the endosomal membrane forms MVBs, and this step enables the cytoplasmic constituents to enter the endosomes and enriches the cargo of the ILVs (future exosomes) [22,23]. According to the needs of the cells, some MVBs can directly fuse with the lysosomes, and the contents of the MVBs will undergo lysosomal degradation and be recycled by the cells. Other MVBs will be transported to and merge with the plasma membrane, eventually resulting in the release of exosomes (Figure 1) [23,24]. 

Exosomes carry several selected subpopulations of RNA and deliver them to interstitial space or bloodstream and these RNAs can be subsequently taken up by, and expressed in recipient cells. Generally, exosomes are enriched in small noncoding RNAs (ncRNAs), including miRNAs, tRNAs, lncRNAs, circRNAs, and fragmented RNAs [25]. These selected RNAs are enriched in specific RNA species relative to the cellular RNA, indicating a specific mechanism associated with exosome biogenesis and content loading [21]. The specific RNA-packaging mechanism has not been fully explained and it may be influenced by the microenvironment and the inherence of the cells. Some studies find the enrichment of miRNAs with 3′-end nucleotide additions and 5′-terminal oligopyrimidine, which indicates the specific modification of RNAs during exosome formation [26,27]. Besides, evidence suggests that several mechanisms may play a part in RNA-packaging of exosomes, with specific subtypes of RNAs bound to different exosome-targeted RNA-binding proteins (RBPs). For example, Shurtleff suggested that Y-box binding protein 1(YBX1), a RBPs with broad nucleic acid binding property, plays a pivotal role in exosomal sorting of miRNAs and other small ncRNAs while other scholars identified the significant contributions of Ago2, a miRNA effector, in RNA-packaging of exosomes [28,29].

Once the exosomes are secreted into the interstitial space or the bloodstream and reach their target cells, they are recognized and enter the recipient cells by different mechanisms. The distinct mechanisms and pathways for exosome uptake are of great complexity. After being docked at recipient cell membranes, some exosomes may fuse with the plasma membrane and release their constituents into the cytoplasm. Some may be taken by recipient cells in multiple pathways, including clathrin-dependent endocytosis, caveolae-dependent endocytosis, macropinocytosis, and phagocytosis [30,31,32]. After internalization of the exosomes, these extracellular vesicles can be degraded by lysosomes or be gathered and coexist with the endogenous ILVs in the MVBs. The exosomal RNAs can be transferred into the endoplasmic reticulum or the cytoplasm and result in the phenotypic and molecular alterations of recipient cell. It should be noted that exosome uptake through phagocytosis and micropinocytosis hamper the delivery of functional RNA into the cytoplasm, as the prominent function of these pathways is to transfer cargos to lysosome for degradation. Thus, some researchers supposed that exosomal RNAs might incorporate some mechanisms to escape the degradative pathway, which so far remain unknown, to elicit a functional response [33]. Future studies may focus on the mechanism and methods of efficiency enhancement of exosomal RNAs escape from lysosomal degradation and widen the application of exosomal RNAs [34]. Like endogenous RNAs, mRNAs delivered by exosomes can be translated into functional proteins, whereas ncRNAs can engage complex networks of ncRNA interactions and serve as important regulators of gene expression. The utilization of these RNAs will activate the subsequent signal pathways and modulate physiological and pathological processes, such as those seen in immune responses, cardiovascular diseases, and cancer. The mode of exosome uptake and the fate of the exosomal cargo varies depending on the property of the cargo and the metabolic status of recipient cells that regulates internalization of extracellular vesicles [35]. However, it remains poorly understood whether a different mode of exosome uptake results in distinct physiological and pathological processes in recipient cells.

## 3. Role of Exosomal RNA in the Initiation and Development of GC

**Figure 2:**
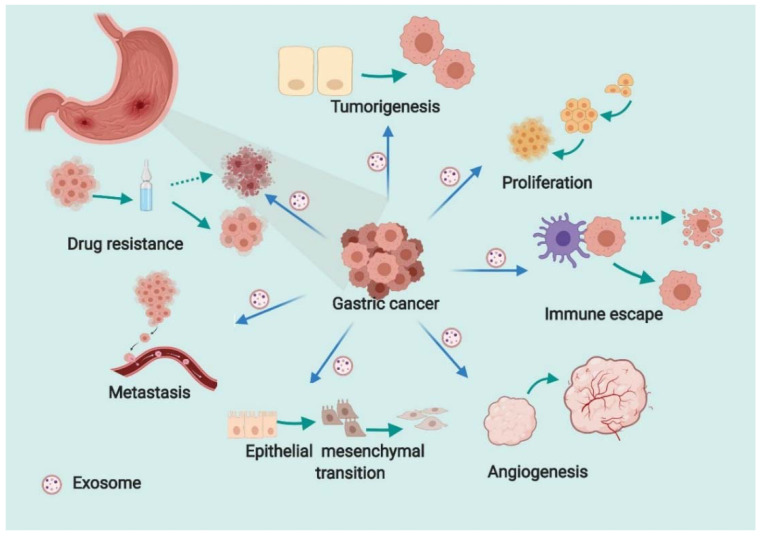
The role of exosomal RNA in the initiation and development of gastric cancer. Exosomes affect tumorigenesis, proliferation, angiogenesis, epithelial–mesenchymal transition, metastasis, immune escape, and drug resistance in GC.

As referred to above, exosomes contain an array of proteins, lipids, DNAs, mRNAs, and ncRNAs, and the exchange of exosomes among cells plays important roles in many aspects of human health and disease. The study of exosomes’ roles in cancer has progressed at a rapid pace, and exosomes have been implicated in several hallmark features of cancers, such as tumorigenesis, tumor growth, epithelial mesenchymal transition (EMT), metastasis, angiogenesis, immune escape, and drug resistance [36,37,38,39,40,41]. In GC, accumulated studies have assessed the efficacy of exosomal contents in the initiation and development of GC. Exosomal RNAs are best investigated due to their powerful role in regulation of gene expression. MiRNAs are a class of ncRNAs with a size of about 20–25 nucleotides, which exert regulatory functions in eukaryotes mainly by interfering translation of mRNA. LncRNAs are a series of ncRNAs with a length of more than 200 nucleotides, which can compete with miRNAs to bind mRNAs or acting together with miRNAs to regulate expression of downstream genes. CircRNAs are another kind of ncRNA formed by the reverse splicing with a covalently closed single stranded circular shape. They can bind to miRNAs and relieve the inhibition of miRNA on target mRNA and, subsequently, increase the expression level of target genes. Despite their distinct mechanisms, they all play important roles in the initiation and progression of GC by manipulating the expression of target genes. We herein summarize the biological function of them in the initiation and development of GC (Figure 2). 

### 3.1. Tumorigenesis

The initiation of GC tumorigenesis is a result of long-term accumulation of gene mutations and functional alteration in TME. Previous reports suggested that exosomes play a pivotal role in the development of various precancerous diseases of GC, the formation of TME and, ultimately, the development of GC. *HP* infection is the single most important factor for GC and recent research has implied that the exosome is implicated in the initiation of *HP*-related diseases. For instance, *HP*-induced exosomal mesenchymal-epithelial transition factor (MET) can exert a pro-tumorigenic effect on tumor-associated macrophages to promote GC progression [42]. Another study indicated that exosomes from conditioned media of human gastric epithelial cells are involved in the endothelial function impairment in *HP* infection [43]. It is also illustrated that exosomal miRNA-155 derived from *HP* infection macrophages can immunomodulate the inflammatory response to inhibit the gastritis [44]. In light of the contribution of *HP* in GC, these studies implicated the function of exosomes in the development of precancerous diseases of GC and ultimately in the initiation of GC. The role of exosomes in the formation and regulation of TME in GC was also explored in the context that exosomes act as the vehicle of information transfer in TME. Adverse conditions like hypoxia, virus infection, and acidosis can increase exosome release of the impaired cells or cancer cells. Those exosomes can induce TME alteration and subsequently facilitate the initiation and progression of GC. Juan indicated that exosomes derived from gastric cancer cells can modulate the immune response in TME to favor the progression of GC [45]. Moreover, oncogenic constituents of exosomes can directly affect the gene expression, transcription, and translation of recipient cells to acquire malignant characteristics. For instance, exosomal miR-101 from normal cells and a tumor suppressor molecule can be secreted into the TME to restrain tumor cells. In the early stage of tumorigenesis, insufficient secretion of miR-101 from residential normal cells may not suppress GC tumor cells and facilitate the initiation of GC [46]. Several other exosomal miRNAs associated with carcinogenesis of GC include exomiR-Let7, exomiR-221, exomiR-25 and exomiR-210 [47,48,49]. In these studies, exosomal RNAs can facilitate the carcinogenesis of GC via the expression of specific proteins. To summarize, exosomes participate in the progression of precancerous diseases of GC, remodeling of TME, and carcinogenic reprogramming to confer the initiation of GC.

### 3.2. Proliferation and Apoptosis 

The rapid growth and expansion of GC leads to the survival of cancer cells and a dismal outcome of chemotherapy. The exosomal RNAs has also been implicated in regulating proliferation and growth of GC though various signing pathways. Hai-Yan manifested that exosomal lncRNA CEBPA-AS1 from GC cells could promote cell proliferation, inhibit apoptosis, and induce GC progression in vivo [50]. Exosomal lncRNA ZFAS1 has been shown to promote GC growth by affecting cell cycles and apoptosis [51]. In these studies, exosomal RNAs participate in the regulation of GC cell proliferation mainly by mediating the rate of apoptosis. Furthermore, recent experiments indicated that exosmal RNAs can affect the growth and expansion of GC by altering the expression of transcription factors or signal pathway proteins. For example, exosomal miR-1290 from BGC-823 cells promotes the proliferation and invasion in gastric cancer via targeting mRNA of naked cuticle homolog 1 (NKD1), a transcriptional regulatory factor in GC and downregulating NKD1 expression [52]. Several exosomal miRNAs are involved in the activation of the MAPK signaling pathway in GC, by which CD97 can enhance proliferation and invasion in vitro [53]. These findings together corroborate the significance of exosomal RNAs in the proliferation and apoptosis of GC cells. Aside from exosomal RNAs, other exosomal cargos, such as proteins or lipids, are also proposed to play a role in GC growth. The delivery of trastuzumab emtansine (T-DM1) from exosomes of HER2-positive cancer cells to other cells has been implicated in growth inhibition and activation of caspase-3 [54]. Another study indicated that the gastric cancer derived exosomes can drive tumor cell proliferation via the activation of PI3K/Akt signaling pathway [55]. This literature together confirms the significance of exosomal RNAs in tumor growth, as well as that of other exosomal contents. It should be noted that the growth and expansion of gastric tumors cannot be attributed completely to the acceleration of cell proliferation, but more so a result of joint action of several processes including apoptosis, angiogenesis, immune escape, and drug resistance, which will be further discussed in the next section.

### 3.3. Angiogenesis

Angiogenesis, essential for tumor growth and metastatic dissemination, is a multi-step process by which tumors develop new vasculature and obtain sufficient nutrition. The function of exosomes in tumor angiogenesis of GC has been recently widely documented. For instance, miR-130a from gastric cancer cells-derived exosomes can enter vascular cells and target C-MYB, a transcription factor of angiogenesis, to promote angiogenesis and tumor growth [56]. MiR-23a carried by GC cells-derived exosomes can be used by vascular cells and suppress the expression of a well-known tumor suppressor gene, PTEN to promote angiogenesis [57]. These exosomal RNAs from TDEs can target vascular cells and promote angiogenesis though expression alternation of transcription factor or tumor suppressor gene. Similar findings implicated that exosomal miR-155 and miR-135b from culture medium of gastric cancer cells also enhanced angiogenesis in GC by inhibiting the expression of transcription factor FOXO3a and FOXO1 [58,59]. In light of the key role of vascular endothelial growth factor (VEGF)/VEGF receptor (VEGFR) signaling pathway in angiogenesis and tumor growth, many studies elucidated that tumor-derived exosomal molecules can induce angiogenesis via directly targeting the VEGF signaling pathway. Mengyan delineated that exosomal circSHKBP1 can sponge miR-582-3p to enhance VEGF mRNA stability and enhance angiogenesis in GC [60]. Ting implied that exosome miR-155 derived from GC targeted the c-MYB/VEGF axis to increase VEGF expression, and subsequently promoted angiogenesis [61]. Moreover, Guangxin indicated that the VEGFR-2 inhibitor can counteract the aggressive behavior of vascular cells triggered by irradiated gastric cancer cells-derived exosomes [62]. The role of exosomes in angiogenesis of GC has been widely documented in recent years, which provides a new orientation to curb angiogenesis and progression of GC. These findings will become more valuable if they can be associated with therapeutic interventions such as chemotherapy. 

### 3.4. Immune Escape

The immune system can monitor, recognize, and eliminate cancer cells and foreign invaders, such as bacteria and parasites. The rapid proliferation and mutation of GC cells can generate different types of antigens, which can be detected, presented, and eliminated by immune innate cells to prevent potentially malignant transformation. However, gastric cancer cells have developed many mechanisms by which they can escape from the surveillance of the immune system and avoid the immune response. These mechanisms include decreased expression of MHC I or MHC II, downregulation of cancer-related antigens, and adhesive molecules [63,64,65,66]. Recently, the exosome was considered to play an active part in this process. TDEs can generate an immunosuppressive environment by attenuating the response of immune effector cells and recruiting immunosuppressive cells. The production of adenosine via the sequential activity of CD39 and CD73 ectoenzymes participates to the generation of an immunosuppressive tumor microenvironment, and it is reported that through the expression of immune-related molecules, such as CD39 and CD73, TDEs can regulate the immune microenvironment of tumor cells and help malignant cells avoid being recognized by immune cells [67,68]. A recent study suggested that TDEs carried Programmed cell death 1 ligand 1 (PD-L1), retain immunosuppressive activity via the downregulation of T-cell surface CD69. Exosomal PD-L1 induced immunosuppression microenvironments and predicted a worse survival rate [69]. Another similar study found that exosomes from GC cell line BGC-823 promoted the PD-L1 expression of neutrophils and suppressed T-cell immunity, which facilitated the immune escape of GC cells [70]. In addition to this, it has been reported that TDEs help cancer cells evade immune recognition by employing decoy mechanisms [67]. Not all studies prefer the promotional effect of exosome in immune escape, in some cases, gene-modified tumor cells can cause the TDEs contain glycosyl-phosphatidylinositol-anchored interleukin 2 (GPI-IL-2), resulting in increased antitumor effects and hamper immune escape [71]. Similar studies enlighten clinical application prospects of exosome-based immunotherapy in cancers. Hitherto, evidences about the exosome role in immune escape are mostly from other cancers like myeloid leukemia, B cell lymphomas, and colorectal cancer and data from GC are relatively rare. However, the cumulative studies above indicated that exosome-mediated immune response count in the immune escape of cancer cells. Thus, a better understanding of the function of exosomes in immune response would be helpful for developing potential exosome-based biomarkers and therapeutics.

### 3.5. Epithelial-Mesenchymal Transition (EMT)

Epithelial-mesenchymal transition (EMT) is defined as the biological process of epithelial cells transforming into mesenchymal cells through specific procedures. During EMT, cells are altered at the molecular level as well as in cell morphology with the loss of polarity, which causes the increased potential for migration. The main hallmarks of EMT is the loss of epithelial E-cadherin and acquirement of mesenchymal markers like vimentin, N-cadherin, and a spindle-like cellular shape [72,73]. Examples of TDEs promoting EMT in GC have been reported in many studies. Generally, exosomes can promote EMT via the following mechanisms: facilitating oncogenic cell transformation, enhancing cell migration and invasion, stimulating angiogenesis, and reprograming the pre-metastatic niche [73]. For instance, Mei demonstrated that exosomal miR-155-5p from Paclitaxel-resistant tumor cells can induce EMT and chemoresistance in sensitive cells [74]. Exosome-transmitted lncRNA PCGEM1 promotes EMT in gastric cancer by maintaining the stability of SNAI1 [75]. MiR-223 was another abundant cargo in GC exosomes which modulate EMT via the PTEN-PI3K/AKT pathway to support cancer promotion [76]. In these studies, exosomal RNAs from tumor cells or TME induce the activation or suppression of several signaling pathway and alter the phenotype and biological behavior of cancer cells, resulting in increased motility, invasiveness, and metastatic potential of cancer cells.

### 3.6. Metastasis

Metastasis is one of the main causes of cancer-related deaths and treatment failure. It is a complex and intricate process that involves several steps including EMT, cancer cell invasion, intravascular transport, and attachment to and engraftment in distant organs. Every step is driven and manipulated by the cooperation of tumors and TME [77,78]. The most common metastatic site and organ of GC is the left supraclavicular lymph node and liver. It is reported that the five-year survival rate of GC patients with metastasis is less than 10% [4]. The biology of exosomes in GC metastasis is emerging, and the number of studies clarifying their function in the above steps has increased substantially. The contribution of exosomal RNA in the metastasis of GC involves all steps of metastasis from EMT, cancer cell invasion, organotropic metastasis, and the formation of the pre-metastatic niche. Firstly, the plasticity of tumor cells can be partly attributed to exosomes, especially considering their significant role in EMT as mentioned above. Next, exosomes were implicated in promoting migration and invasion ability of GC cells via various signaling pathways. Exosomal miR-196a-1 from high-invasive GC cells can target secreted frizzled related protein 1(SFRP1), the modulator of Wnt signaling, in low-invasive cells to promote invasion and metastasis [79]. Moreover, recent experiments have also indicated a function of exosomes in facilitating the landing of metastatic cancer cells and the formation of a pre-metastatic niche. Exosomal EGFR was reported to effectively remodel liver microenvironments via suppressing miR-26a/b expression to favor gastric cancer liver metastasis [80]. RNAs in TDEs can activate alveolar epithelial toll-like receptor 3 (TLR3) to promote neutrophil recruitment and confer lung premetastatic into a niche formation [81]. Thus, exosomal RNA can participate in most processes of GC metastasis, promote the progression of GC, and pose more challenges to GC treatment.

### 3.7. Drug Resistance

Chemotherapy is considered the most effective treatment modalities for GC patients, and drug resistance remains the greatest challenge for antitumor therapy [82]. The mechanism of drug resistance is complicated, among which function of exosomes counts. In normal cells, exosomes function in the transfer of abundant cargos into the TME. In the cancer context, some “anti-chemotherapy” information may be packed in exosomes, released into the TME, and eventually deciphered by other cancer cells. The transmission of those message may endow the sensitive cancer cells with a drug resistance ability, which is revealed by accumulated evidence. A recent study indicated that exosomal miR-21 derived from tumor-associated macrophages confers cisplatin resistance in GC [83]. Haiyang suggested that CAFs secrete miR-522 to suppress ferroptosis by targeting arachidonate lipoxygenase 15(ALOX15) and promote acquired chemoresistance in gastric cancer [84]. Moreover, some researchers have identified many other exosomal RNAs, like lncRNA HOTTIP, miR-501, miR-106a-5p, and miR-421, which could be involved in the chemoresistance of GC [85,86,87]. Under these circumstances, exosomal RNAs aid in the acquisition and spread of drug resistance property and limit drug activity toward cancer cells. Besides, chemotherapy and radiation therapy could also directly affect exosome biogenesis and the content of exosomes with potential implications on therapy outcome. It should be noted that the function of exosomal RNA in drug resistance is complicated and some scholars claim that exosomal RNAs may also play a role in reducing drug resistance [88]. Apart from chemoresistance, exosomes are also found to be of great value in determining outcomes in radiation therapy and some studies suggested that exosomes may induce the radiation-induced bystander effect (RIBE) [89]. Since radiotherapy is seldom conducted in the treatment of GC, reports about the role of exosome in GC radiotherapy resistance is relatively few. Building on the observation that exosomal RNAs play an active part in the mechanism of drug resistance, engineering of exosomes to deliver a specific RNA for GC will be developed in the near future.

## 4. Clinical Application of Exosomal RNAs in GC

### 4.1. Diagnostic Potential of Exosomal RNAs as Biomarkers of GC

One feature of GC is the lack of specific manifestations in the early stage, which make early detection difficult and may delay the optimal treatment period [90]. Currently, endoscopy combined with biopsy is considered the gold standard for GC diagnosis. Endoscopy is an invasive examination with a relatively high cost; hence, a novel non-invasive diagnostic method is urgently needed. Liquid biopsy has emerged as a non-invasive approach with the potential to identify tumor related biomarkers. Recently, researchers have made great efforts to identify tumor-derived components, such as circulating tumor cells (CTCs), circulating tumor DNA (ctDNA), serum miRNAs, and exosomes for a diagnostic purpose [91,92,93,94,95]. 

Previous studies have indicated that the exosomal RNAs outperform plasma miRNAs in the reflection of cancer progression and the early diagnosis of cancers with the following advantages: the miRNAs in exosomes can be protected from being degraded by RNase; the content of exosomes is closely related to that of donor cells which confers the exosome-based detection a higher specificity; and the concentration of exosomes in body fluid is higher than traditional markers [96,97,98]. In this section, we discuss the state-of-the-art exosomal RNAs in GC.

Among those exosomal molecules, miRNAs are considered the most potentially ideal marker for its abundance and easy accessibility. Recently, the potential value of miRNAs as biomarkers for early diagnosis and prognostic prediction of GC has been reported. For instance, Ning et al. found that the levels of miR-19b and miR-106a in exosomes of patients with GC were markedly overexpressed compared to healthy subjects, indicating that serum exosomal miR-19b-3p and miR-106a-5p had the potential to aid in the detection of GC [99]. However, the authors of this study did not explore its value in the early detection of GC. Some exosomal miRNAs such as miR-1246, miR-92b-3p, let-7g-5p, miR-146b-5p, and miR-9-5p were found to be significantly associated with early-stage GC and may serve as early GC biomarkers [100]. Additionally, the expression of some exosomal miRNAs was corelated with lymph node metastasis and tumor stage, or was proposed as independent prognostic factors for GC. For example, Huan et al. suggested that exosomal miR-423-5p increased in GC patients, and the elevated exosomal miR-423-5p was significantly associated with lymph node metastasis and indicated a poor outcome [101].

Existing studies on lncRNAs remain limited compared to those on miRNAs. Recently, studies validated that exosomal lncRNAs can also serve as tumor biomarkers for GC. Chenchen suggested that the exosomal expression of LncRNA PCSK2-2:1 of GC patients was markedly downregulated compared to the control group, and was correlated with tumor size, stage, and venous invasion [102]. This study and researches alike validated the possibility to use lncRNAs to predict metastasis and prognosis of GC. Another study indicated that the diagnostic value of exosomal lncRNA SLC2A12-10:1 in discriminating GC patients from healthy subjects was higher than traditional tumor biomarkers, which highlighted the potential utility of this exosomal lncRNAs as novel tumor markers for GC screening [103].

circRNAs are another kind of endogenous ncRNAs which may act as tumor markers in GC. Recently, circRNAs has become a new hotspot in the field of tumor research. More and more attention has been paid to its potential value in tumor diagnosis, treatment, and prognosis evaluation. To date, the relationship between exosomal circRNAs and tumor diagnosis mainly focuses on lung cancer and breast cancer, and reports regarding GC are, at best, sparse. A recent study suggested that circPVT1 expression was an independent prognostic marker for overall survival and disease-free survival time of GC patients [104]. It has been reported that an obvious decrease was observed in the exosomal hsa_circ_0065149 levels of early GC patients compared to a healthy group and expression of ciRS-133 derived from GC patients’ serum was significantly higher than the control group [105,106]. These studies offered the promise that exosomal circRNAs may also become novel diagnostic markers for GC diagnosis and prognosis evaluation in the near future.

As mentioned above, different exosomal RNAs including miRNAs, LncRNAs, and circRNAs are described as potential GC diagnostic and prognostic biomarkers, which are summarized in Table 1. Although these exosomal RNAs were described as potential GC biomarkers, there is still a need for experiments to corroborate their value in the early detection of GC. The need for sensitive and specific exosomal biomarkers will continue to grow as our knowledge of the exosome grows. There may be a long way to go before finding a highly sensitive, stable, and non-invasive GC biomarker. 

### 4.2. Therapeutic Potential of Exosomes in the Treatment of GC

Since GC ranks as the fourth most common cause of cancer-related death in the world, it is imperative to seek better targeted therapies. In light of their characteristic property in delivering functional molecules to targeted cells, exosomes may serve as therapeutic vehicles of GC therapy, both at the basic and applied levels. Recently, exosomes designed for the delivery of therapeutic agents are being actively explored [124,125,126]. Compared with previous drug carriers such as liposomes, exosomes have the following advantages: they are efficient at penetrating biological barriers and entering other cells; the heterogeneity of exosomal surface molecules favor their receptor-targeted feature and make targeted therapies for cancer possible; and they are well tolerated and can deliver therapeutic agents with minimal immune clearance [127,128]. Therefore, the therapeutic application of exosomes as nanocarrier is promising. In this section, we introduce the advancements in research of the therapeutic potential of exosomes in GC.

Over the last few years, great efforts have been made to engineer exosomes for the encapsulation of therapeutic agents such as miRNAs or its inhibitors. Usman proved that EVs can serve as a versatile delivery system for therapeutic RNAs in leukemia and breast cancer cells [129]. In GC, researchers found that macrophage-secreted exosomes can transfer miRNA-21 inhibitors into cancer cells and regulate the proliferation and migration ability of recipient cells [130]. Another study suggested that exosomes containing hepatocyte growth factor siRNAs can inhibit the proliferation and migration of both cancer cells and vascular cells in GC [131]. This progress provides preliminary evidence for the application of exosome in intervention of GC progression in vitro. Furthermore, some scholars corroborated that exosomes can not only be engineered to regulate tumor growth or migration but also play a role in the solution of drug resistance. Wang illustrated that exosomes can act as the vehicle to deliver anti-miR-214 to GC cells and reverse chemoresistance to Cisplatin [132]. Qiumo proved that exosomal c-Met siRNA can significantly restrain the aggressive behavior of GC and partly reverse the chemoresistance to Cisplatin in vitro and in vivo [133]. Together, these preclinical studies offer encouragement for the application of exosomes as vehicle of therapeutic agents.

Despite the promising prospect and these pioneering works, the application of exosomes as a nanocarrier in clinical treatment of GC still has many difficulties to overcome. To begin with, it is hard to guarantee the homogeneity of exosomes. While some types of exosomes present the potential of tumor inhibitory effects, others may not have therapeutic effects, or may even have the opposite effects to facilitate tumor progression. Then, current isolation methods of exosomes are relatively inefficient and cannot satisfy the requirement of immunotherapy. Anyway, we cannot ask an infant to do everything well. As more and more clinical research and improvement of exosomes extraction techniques, exosomes will shine brilliantly in the clinical treatment of GC.

## 5. Conclusions and Perspectives

As outlined above, exosomes are reported to be internalized and mediate the activity of recipient cells, thereby functioning as vehicles of intercellular communication. In this review, we introduced many cargos of exosomes including miRNAs, lncRNAs, and circRNAs, which function in hallmarks of GC. These findings may enlighten more studies regarding the pathogenesis and mechanism of GC, hopefully opening up new avenues in the treatment of GC. However, the question remains whether such phenotypic and molecular alterations are of relevance because of the use of supra physiological amounts of cell culture-derived exosomes in most studies. More precise studies and characterization procedures are needed to verify its function in physiological status.

The cargos of exosomes present the features of donor cells and are preserved in a relatively independent environment with a high stability. This allows for a multicomponent diagnostic window into disease detection and monitoring. However, large-scale studies are urgently needed to ascertain their value before the application of those exosomal constituents as highly sensitive, stable, and non-invasive biomarkers of early GC. The property of exosomes in delivering functional molecules to target cells also advances their potential utility as therapeutic vehicles, both at the basic and applied levels. In some studies, exosomes are engineered to deliver therapeutic agents and direct their delivery to a specific target. Most of these studies are in a pre-clinical experimental stage, and there is still a long way to go before the practical applications of exosomes can become an effective treatment strategy.

## Figures and Tables

**Table 1:** Potential exosomal RNA biomarkers in gastric cancer.

Biomarker Type	Molecules	Exosome Origin	Stage of GC	Description/Function/Importance	Ref.
miRNAs	miR-423-5p	serum	Stage I-IV	The expression of exosomal miR-423-5p was correlated with lymph node metastasis.	[101]
	miR-19b-3p miR-106a-5p	serum	NA	The higher expression was related to GC lymphatic metastasis and was observed in stages III and IV compared to I and II stages.	[99]
	miR-23b	plasma	Stage I-IV	An independent prognostic factor for OS and DFS at each GC stage.	[107]
	miR-21 miR-92a	plasma	Stage II and III	Independent prognostic factors for OS and PRFS in stage II and III GC.	[108]
	miR-181b-5p	ascites	Stage III-IV with ascites	Distinguished between non-malignant and GC-ascites.	[109]
	miR-374a-5p	serum	20% from stage I-II and 80% from stage III-IV	Upregulation predicted poor prognosis.	[110]
	miR-379-5p miR-410-3p	serum	37% from stage II and 63% from stage III	Higher expression indicated shorter progression-free survival of the patients.	[111]
	miR-92b-3p, let-7g-5p, miR-146b-5p, miR-9-5p	serum	Stage I–II	Higher levels of those serum exosomal miRNA were significantly associated with early stage GC.	[100]
	miR-221	Peripheral blood	NA	Positively correlated with poor prognosis	[112]
	miR-10b-5p, miR-101-3p, miR-143-5p	plasma	Stage I–IV	Proposed as biomarkers for lymph node metastasis, ovarian metastasis, and liver metastasis, respectively.	[113]
	miR-21-5p, miR-92a-3p, miR-223-3p miR-342-3p	peritoneal fluid	NA	Positively correlated with peritoneal cancer index.	[114]
	miR-1246	serum	Stage I	Differentiated GC patients with TNM stage I from healthy controls and patients with benign diseases.	[115]
	miR-217	serum	NA	Enhanced gastric cancer cell proliferation, and reduced exosomal CDH1 level.	[116]
	miR-1307-3p	serum	NA	The expressions were significantly increased in GC group.	[117]
lncRNAs	lncRNA-GNAQ-6:1	serum	43% from stage I–II and 57% from stage III–IV	Expression was significantly lower in the gastric cancer group.	[118]
	lncRNA-GC1	serum	46% from stage I–II and 54% from stage III–IV	LncRNA-GC1 may serve as a noninvasive biomarker for detecting early-stage GC and for monitoring disease progression.	[119]
	lncRNA PCSK2-2:1	serum	37% from stage I–II and 63% from stage III–IV	Expression level in GC patients was significantly downregulated and was correlated with tumor size, tumor stage, and venous invasion.	[102]
	lncRNA HOTTIP	serum	44% from stage I–II and 56% from stage III–IV	Expression levels were typically upregulated in GC and were significantly correlated with invasion depth and TNM stage.	[86]
	lncUEGC1	serum	Stage I	AUC values of 0.8760 and 0.8406 in discriminating early GC patients from healthy individuals and chronic atrophic gastritis, respectively.	[120]
	lncRNA MIAT	serum	NA	Serum exosomal MIAT levels were significantly higher in GC patients than in gastric adenoma patients and healthy controls, and may serve as a promising novel biomarker for monitoring the progression of GC.	[121]
	lnc00152	plasma	43% from stage I–II and 57% from stage III–IV	Significantly elevated in GC patients compared with healthy groups.	[122]
	RNA H19	serum	49% from stage I–II and 51% from stage III–IV	Significantly upregulated in patients with GC both before and after surgery, and preoperative lncRNA H19 levels were significantly correlated with the TNM stage.	[123]
	CEBPA-AS1	plasma	Stage I–IV	The AUC value of CEBPA-AS1 was higher than those of other traditional tumor biomarkers	[100]
circRNAs	Hsa_circ_0065149	plasma	25% from stage I–II and 75% from stage III–IV	Significantly decreased in early GC patients, and has higher sensitivity and specificity than traditional clinical biomarkers.	[105]
	ciRS-133	plasma	NA	Markedly higher compared to normal subjects.	[106]

Abbreviations: micro RNA (miRNA or MiRNA); circular RNA (circRNA); long non-coding RNA (lncRNA); disease-free survival (DFS); overall survival (OS); area under the curve (AUC); Not available (NA).

## Data Availability

No new data were created or analyzed in this study. Data sharing is not applicable to this article.
